# Visual Exploration and Object Recognition by Lattice Deformation

**DOI:** 10.1371/journal.pone.0022831

**Published:** 2011-07-27

**Authors:** Vasile V. Moca, Ioana Ţincaş, Lucia Melloni, Raul C. Mureşan

**Affiliations:** 1 Department of Experimental and Theoretical Neuroscience, Center for Cognitive and Neural Studies (Coneural), Romanian Institute of Science and Technology, Cluj-Napoca, Romania; 2 Department of Neurophysiology, Max Planck Institute for Brain Research, Frankfurt am Main, Hessen, Germany; National Institute of Mental Health, United States of America

## Abstract

Mechanisms of explicit object recognition are often difficult to investigate and require stimuli with controlled features whose expression can be manipulated in a precise quantitative fashion. Here, we developed a novel method (called “Dots”), for generating visual stimuli, which is based on the progressive deformation of a regular lattice of dots, driven by local contour information from images of objects. By applying progressively larger deformation to the lattice, the latter conveys progressively more information about the target object. Stimuli generated with the presented method enable a precise control of object-related information content while preserving low-level image statistics, globally, and affecting them only little, locally. We show that such stimuli are useful for investigating object recognition under a naturalistic setting – free visual exploration – enabling a clear dissociation between object detection and explicit recognition. Using the introduced stimuli, we show that top-down modulation induced by previous exposure to target objects can greatly influence perceptual decisions, lowering perceptual thresholds not only for object recognition but also for object detection (visual hysteresis). Visual hysteresis is target-specific, its expression and magnitude depending on the identity of individual objects. Relying on the particular features of dot stimuli and on eye-tracking measurements, we further demonstrate that top-down processes guide visual exploration, controlling how visual information is integrated by successive fixations. Prior knowledge about objects can guide saccades/fixations to sample locations that are supposed to be highly informative, even when the actual information is missing from those locations in the stimulus. The duration of individual fixations is modulated by the novelty and difficulty of the stimulus, likely reflecting cognitive demand.

## Introduction

The investigation of object recognition in the human visual system is a challenging problem and often requires visual paradigms able to manipulate various features of the stimulus in order to increase or decrease the ability of human subjects to detect, categorize, or precisely identify a target object. Most present methods do not allow for a precise control over the information that is provided to the visual system because they allow multiple features to be present in the image, thereby making it very hard to manipulate how much a given feature contributes to the recognition process. Ideally, one would have a single feature present in the target image and devise a method to manipulate – in a precise quantitative fashion – how much information that feature conveys about the identity of the object. Based on their ability to isolate visual features, techniques to manipulate object perception can be divided into two major categories: *transformative* and *generative*.

We call *transformative techniques* methods where the stimulus is the original image or is created directly from the original image of the object via some image transformation (adding noise, phase spectrum or contrast manipulation, and so on). The fully visible stimulus always consists of the original image of the object. Example techniques include image degradation [1], degradation based on Gaussian filters [2], morphing [3], manipulation of contrast either directly [4] or using controlled agglomerations of pixels [5]. Many of these methods however do not preserve low-level features of the stimulus such as contrast, global luminance, or distribution of spatial frequencies. It has been suggested that high-level object perception must be isolated from low-level processes dependent on image properties because the latter can create confounds in the investigation of object recognition [6], [7]. Therefore, ideally, transformations of the original image need to preserve low-level image properties as much as possible. Methods that achieve this also exist. For example, Tjan et al. [8] manipulated signal-to-noise ratio (SNR) by mixing the image with pink-noise but keeping the mean luminance and the root mean squared (RMS) contrast constant. Another powerful technique is random image structure evolution (RISE), which manipulates the phase spectrum (via a continuous transformation from original to some other phase spectrum of choice, such as a random phase spectrum) in such a way that the global luminance, the contrast and the distribution of spatial frequencies are preserved [7], [9]–[11].

*Generative techniques* create the stimulus indirectly, i.e., information from the original image is used to generate a novel stimulus image from various sets of basic elements (dots, color patches, line segments, Gabors). As a result, the original image or transformed versions of it are not present in the stimulus directly but only some features (e.g., patterns or contour) are conveyed by the basic elements used to render the stimulus. For example, a “Dalmatian Dog” can be represented embedded into a set of distractors [12] by using only patterns of black and white color patches. However, in this case it is difficult to smoothly manipulate the visibility of the object across a given scale. For this reason, other techniques rely on Gestalt principles, such as good continuation or grouping, which can be continuously manipulated. Popular methods use a field of oriented Gabors where the identity of an object can be revealed by forming a more or less coherent contour. The coherency of the contour, and thereby the detectability/identifiability of the object, can be manipulated by progressive alignment of randomly oriented Gabors to the contour of the target object [13]–[15]. Another possibility is to use a field of randomly oriented Gabors and to progressively align the orientation of a local group of Gabors such that it represents a solid shape rather than its contour [16]. In this latter case the representation of the shape relies on the Gestalt principle of grouping by similarity.

Importantly, for many transformative techniques local contrast or luminance may change dramatically by manipulating image structure (e.g. via the phase spectrum) even if global properties are kept constant. Also, these methods do not generally allow for a precise control over the type of information that is provided to the visual system because, by their nature, they allow multiple features (local contours of various spatial frequencies, texture, shading, etc) to be present in the image. Some of the transformative techniques can be improved to solve part of these problems (for example it is straightforward to control spatial frequency information in the RISE method). By contrast, generative techniques suffer less from these problems albeit with the tradeoff of being able to convey only a limited type of visual information. As an example, manipulation of contour by local Gabor orientations keeps both global and local image properties constant, such as luminance and contrast, but the method is able to convey only contour information. The limitation of visual features is desirable for gaining good control over the information conveyed to subjects but may prove overly restrictive for some tasks, such as subordinate-level categorization. In addition, generative techniques are relatively scarce and sometimes require extensive preprocessing of source images. For example, contour manipulation via oriented Gabors needs source images where relevant contours of objects have already been isolated, i.e. sketch images.

Both transformative and generative techniques have been used to study the role of top-down modulation in object recognition because they are able to manipulate object-related information content of the stimulus in order to control the ability of subjects to recognize objects. A popular design to study top-down modulation of recognition involves perceptual hysteresis [5], i.e., a drop in detection or recognition threshold when subjects have been previously exposed to targets as compared to the situation where subjects are naive. Perceptual hysteresis can be studied by progressively manipulating recognizability of the target, first in an ascending fashion (from difficult to easy), where the subject is naive, and subsequently in a reversed, descending fashion (from easy to difficult), where the subject has already been exposed to clear targets and therefore possesses prior knowledge about them [5], [17]. As a result of this priming, detection or recognition accuracy is increased during the descending presentation of targets, presumably because top-down influences facilitate object perception [5], [17]. For obvious reasons, the above mentioned techniques to produce stimuli are useful to study perceptual hysteresis because they can manipulate stimulus recognizability in a parameterized way. Another technique frequently used in the study of perceptual hysteresis is masking where the stimulus is flashed for a limited duration and followed by a mask [18], [19]. Unlike transformative and generative techniques, masking does not require that the stimulus is changed to modulate its recognizability but renders recognition difficult because of the limited access to the stimulus. This makes it suitable to use original, unprocessed images, although masking is frequently used also in combination with stimuli produced via transformative [7] or generative methods [15]. We consider that masking is a less natural way of studying object perception and perceptual hysteresis because it prevents free visual exploration. Since most studies have used stimuli in combination with masking, little is known about how perceptual hysteresis is manifested under naturalistic, free viewing conditions.

Here we set out to develop a novel generative technique, called the “Dots” method that facilitates the study of free visual exploration during object recognition. We applied the “Dots” method to study integration of visual information by human subjects and to investigate how perceptual hysteresis is manifested during free visual exploration.

## Results

### The “Dots” method

The “Dots” method was designed to enable the experimenter to precisely control the amount and type of object-related information provided to the subject. The method exploits several Gestalt principles such as grouping by proximity and good continuation. Visual stimuli are generated by the controlled deformation of a lattice of dots that is driven by a single feature of the original image: local contour density. The method creates a map of points of interest (POI) to compute local information content (local contour density) around each pixel in the source image. This computation is similar to assigning a “saliency” value to each pixel in the source image [20]. To generate the stimulus, a lattice of dots with square structure [21] is then progressively deformed based on the POI map such that dots converge towards corresponding salient regions in the source image. Local contour density information from the source image is therefore progressively represented by the lattice. By adjusting the amount of deformation, the stimulus from the source image can be more or less visible in the deformed lattice.

**Figure 1:**
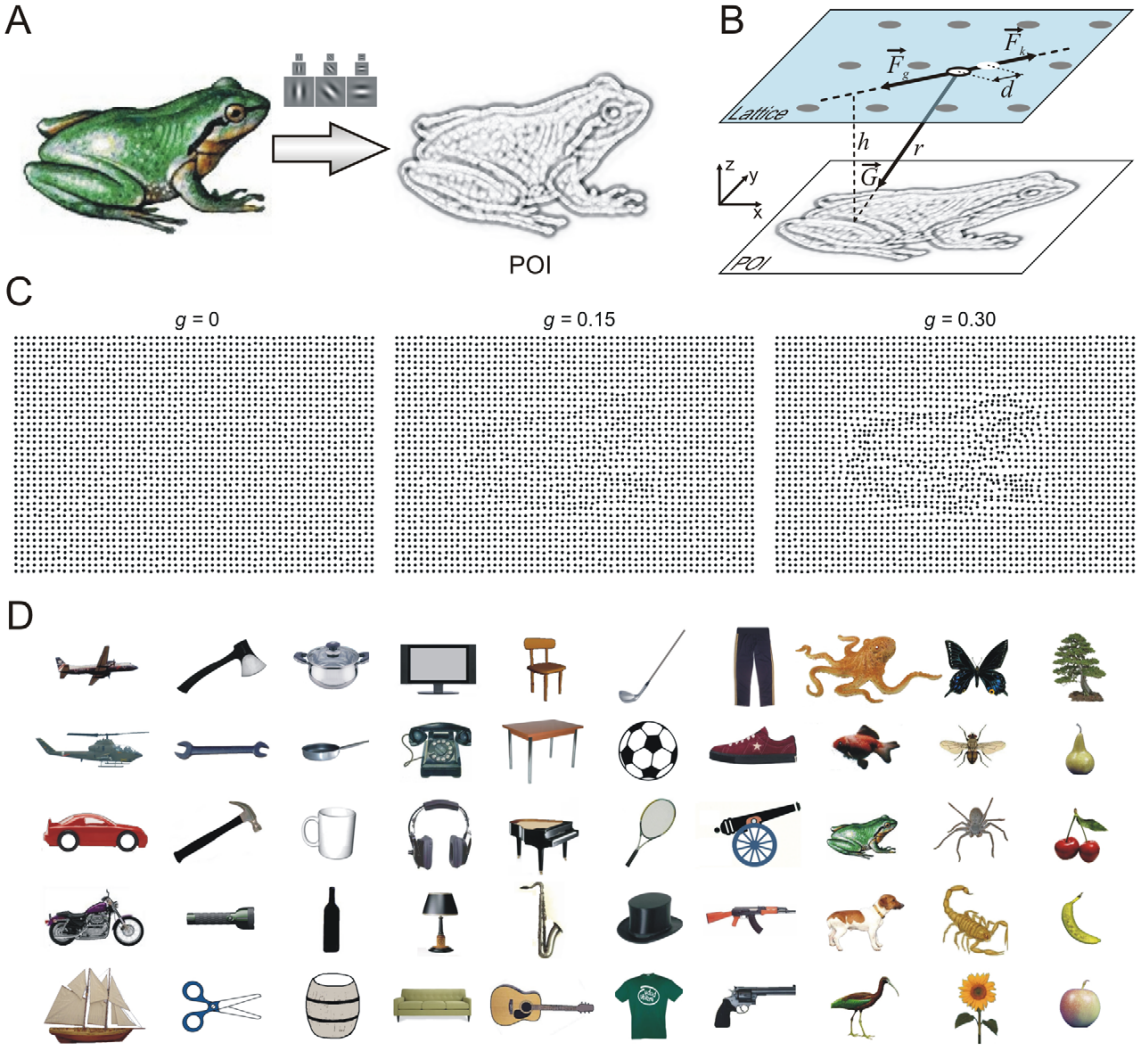
Stimulus creation. (A) The source image is filtered with Gabor wavelets to yield a map of points of interest (POI) estimating the local information in the source image (dark points are most informative). (B) Dots of an elastic lattice are attracted towards points in the POI plane by the projection *F_g_* of a gravitational force *G*; an elastic force, *F_k_*, limits their displacement. Dot movement is confined to the “Lattice” plane. (C) Progressive deformation of a lattice of dots as *g* is increased. (D) Set of objects used to test the method on human subjects.

The POI map computes the local information around pixels in the original image (first converted to grayscale) by applying a local Gabor Wavelet (Gabor Jet) decomposition (Figure 1A) around each pixel [22]. We used Gabor Jets composed of a set of zero-mean Gabor filters spanning a range of spatial frequencies (0.1, 0.25, and 0.5 cycles/pixel implemented as convolution kernels with Gabor sigma of 1.16, 2.16, and 4.83 pixels, respectively, and spanning 7, 13, and 29 pixels, respectively) and different orientations (0°, 30°, 60°, 90°, 120°, and 150°). Each Gabor filter in the jet is convolved with the local image around the pixel and yields a local response. The sum of responses across the jet reveals the importance of the respective pixel in the source image. The POI map can be interpreted as a map describing the contour information content of the original image estimated locally around each pixel. Points located close to contours with higher local contrast or at the intersection of multiple contours will have a stronger response in the summed responses of local filters than points located close to surfaces with smooth luminance changes. Thus, the POI map uncovers the most important points required to identify an object by its representative local contours.

The method employs two parallel plane surfaces (*xy* coordinates) located at a distance *h* (Figure 1B). One plane consists of the POI map, normalized to contain values between 0 and 255. The other plane is a regular lattice of dots. Each pixel in the POI map exerts a gravitational attractive force upon lattice elements proportional to its information content; lattice elements resist movement with an elastic force. Consider one pixel from the POI map and a lattice element situated apart at distance, *r*, (Eq. 1). A gravitational force, *G*, (Eq. 2) attracts the lattice element towards the position of the pixel in 3D space (Figure 1B). Each lattice element has a generic mass, *m_L_*, and each pixel in the POI map has a mass, *m_P_*, directly proportional to its local information content (sum of filter responses across the Gabor Jet). The movement of lattice elements is restricted to the lattice plane and thus only the projection of *G* on the lattice plane, *F_g_*, acts on lattice elements (Eq. 3). Assume that the lattice element has already been pulled by gravitational forces and lies at distance, *d*, (Eq. 6) from its original position. At this position, an elastic force, *F_k_*, proportional to the displacement (Eq. 7) acts like a spring that pulls the lattice element back towards its initial position.
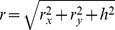
(1)

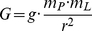
(2)

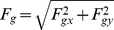
(3)

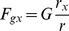
(4)

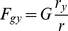
(5)where, *h* is the distance between the POI plane and the lattice plane, *r* is the distance between the POI and the lattice element; *r_x_* and *r_y_* are projections of the vector distance *r* onto the *x* and *y* axes of the lattice plane; *G* is the gravitational force that pulls one lattice element towards a POI; *g* is the gravitational constant; *m_P_* is the mass of the pixel in the POI map (proportional to the energy of the corresponding Gabor jet); *m_L_* is a constant value for the mass of lattice elements (we fixed it to a value of 1); *F_g_* is the projection of the gravitational force *G* on the lattice plane, *F_gx_* and *F_gx_* are projections of *F_g_* on the *x* and *y* axes.
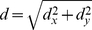
(6)

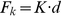
(7)

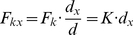
(8)

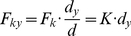
(9)where, *d* is the distance of the lattice element from its original position, *d_x_* and *d_y_* are its projections on the *x* and *y* axes; *F_k_* is the elastic force, *F_kx_* and *F_ky_* are its components on *x* and *y* axes; *K* is an elastic constant.

The deformation of the lattice is solved by an iterative algorithm. At each step, for each lattice element the resultant force is computed as the superposition of gravitational forces from all POI pixels and the corresponding elastic pull. The movement on both *xy* directions is then directly proportional to the resultant forces (Eqs. 10, 11). The iterative process stops either when lattice elements have stabilized, or when a certain number of iterations have been performed.
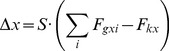
(10)

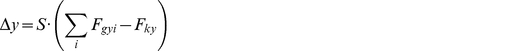
(11)where, *i* indexes all POI pixels; Δ*x* and Δ*y* are the movements of one lattice element on the *x* and *y* directions respectively; *S* is a scaling constant (here a value of 5) that converts the forces into displacement.

The entire process is controlled by three parameters. First, the elastic constant, *K*, (Eq. 7) restricts the movement of lattice elements. Second, the gravitational constant, *g*, (Eq. 2) controls the strength of the gravitational forces and determines how much the grid is deformed to represent the information in the original image (Figure 1C). Third, the distance, *h*, between the two planes controls how diffuse are the gravitational forces (Eqs 1, 2) and ensures a minimum distance between POIs and lattice elements thereby limiting gravitational forces and ensuring convergence of the iterations (Eqs 1, 2). In practice, *K* and *h* are fixed to some satisfactory values and only *g* is then varied to manipulate visibility. Thus, the “Dots” method enables relevant visual information about objects in the source image to be transferred into the stimulus in a controlled fashion.

### Behavioral data

To study object detection and explicit object recognition during free visual exploration, we used stimuli generated with the “Dots” method in psychophysical experiments. Images of 50 objects without background (Figure 1D) were used to create 7 stimuli for each object, with visibility level ranging from no visibility (*g* = 0; no information from the source image was transferred into the dot lattice) to easily visible (*g* = 0.3; where subjects could easily identify the object from the source image) (see Materials and Methods, Stimuli). Depending on whether reaction times were stressed as being important or not, two different experiments were carried out (see Materials and Methods, Experiments). In the first experiment, instructions given to subjects emphasized accuracy but mentioned that speed was important as well, while in the second experiment subjects were given no instructions regarding response speed. Twenty six subjects participated, 14 in the first experiment and 12 in the second experiment (see Materials and Methods, Subjects). Subjects were allowed to visually explore each stimulus for as long as they wanted. They were instructed to decide whether the dot pattern represented something meaningful by indicating with a separate keypress whether they perceived “Nothing”, they saw something but were “Uncertain” what it was, or they have “Seen” a clear object (see Materials and Methods, Procedure). After the button press, participants were required to verbalize their response and also the name of the object, when this was the case.

The psychophysical experiments relied on the ability of the “Dots” method to precisely control recognition at threshold, such that the middle ground between detection (signaling the presence of an object without the ability to identify it) and explicit recognition could be thoroughly investigated. Importantly, detection and explicit recognition were not disentangled by limiting exposure duration [19] but by limiting the amount of available object-related information while allowing the subjects to freely explore stimuli, for unlimited duration. This strategy enables the brain to explore visual solutions and to incrementally integrate visual information to reach a decision. In addition, previous research has shown that recognition can be affected not only by the visibility of a stimulus, but also by previous exposure to it (i.e., perceptual priming [5]; see also [23], [24] for reviews), and that such effects have measurable fMRI/EEG correlates [17], [25], [26]. Therefore, in addition to manipulating stimulus visibility, we also attempted to determine how recognition-related aspects (performance, visual exploration of the stimulus) are affected by the stimulus presentation strategy (order of presentation of stimuli with different visibility levels) and thus the ensuing effect of top-down processes. Using a between-subjects design, two versions of the task (conditions) were run for each of the two experiments (see Materials and Methods, Experimental design). Stimuli obtained from the 50 source images and with the same visibility level (same *g* value) were grouped in blocks, each block corresponding to a different visibility level. In one condition, blocks were presented in an ascending order of visibility (from *g* = 0 to *g* = 0.3), corresponding to a naive subject that had no prior information about the identity of the objects before reaching recognition threshold. In the second condition, blocks were presented in reverse order of *g* value (from *g* = 0.3 to *g* = 0), such that when approaching recognition threshold from above, subjects were already primed by previous exposure to the fully visible objects.

Dependent variables of interest were (1) percentage of subjects' button-press responses for each response type (“Nothing”, “Uncertain”, “Seen”), (2) verbal response accuracy (percentage incorrect, “Uncertain”, correct), and (3) reaction time (RT). A verbal response was considered correct if the subject correctly identified the object at a basic or subordinate level, or if the subject responded “Nothing” to a stimulus with *g* = 0. Since dot stimuli do not allow for fine details to be discriminated, in one case we also considered a response correct if the subject-given label was of a structurally similar object (e.g., “hand mirror” instead of “tennis racket”). A verbal response was considered incorrect if the subject assigned the wrong label to a stimulus, or if he/she responded “Nothing” at *g*>0. “Uncertain” responses were not considered as either correct or incorrect, but were treated as a separate category. Because all psychometric curves, except for reaction times, were very similar in the two experiments we collapsed the former across experiments.

**Figure 2:**
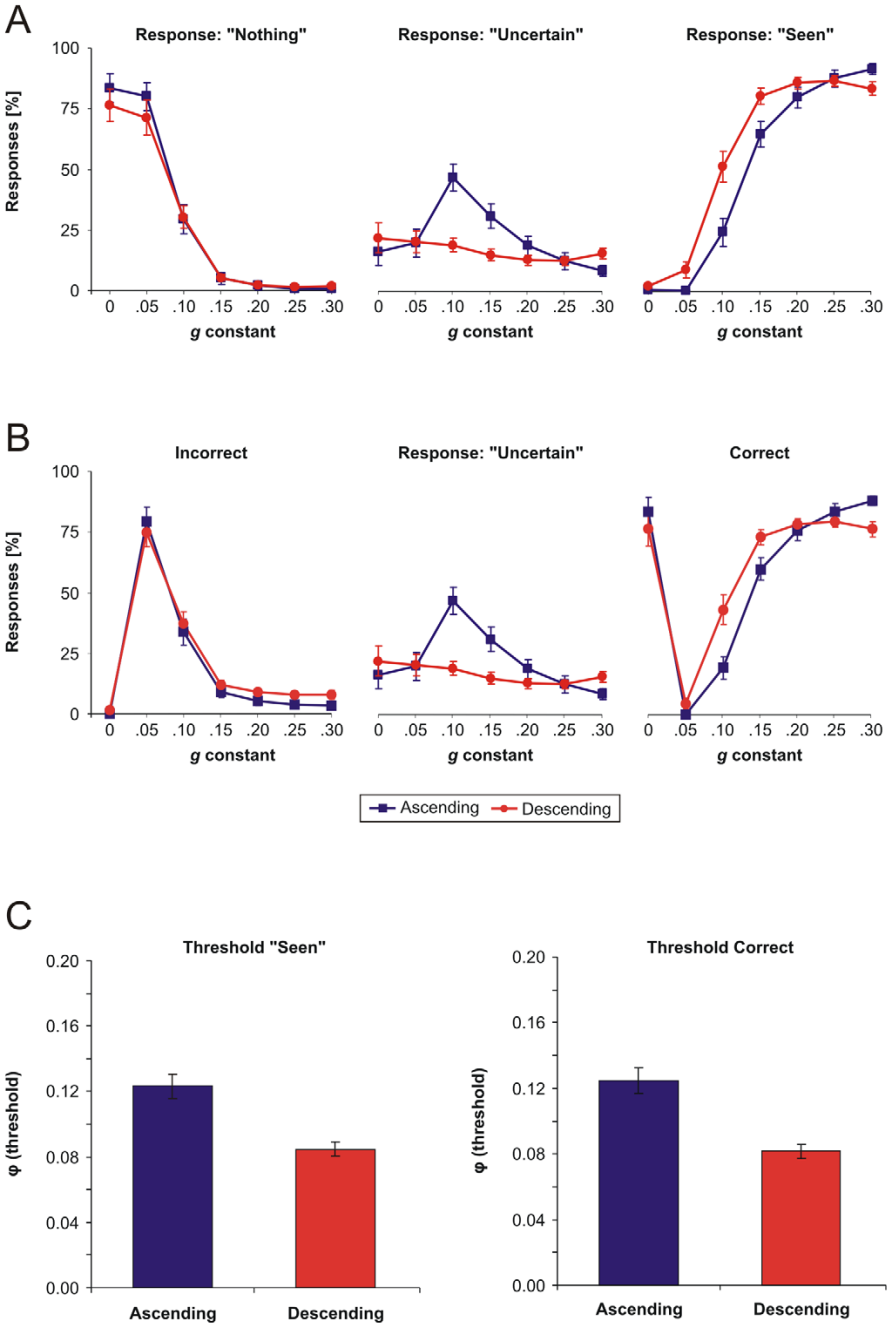
Psychometric curves, subjective and objective thresholds. Psychometric curves grouped by response type (A) and by verbal report accuracy (B) as a function of visibility (*g* value) and experimental condition (ascending and descending). (C) Thresholds for sigmoidal response curves corresponding to “Seen” (subjective) responses (left) and correct (objective) verbal responses (right). Error bars represent s.e.m.

We first computed the percentage of responses as a function of response type for both the ascending and the descending experimental conditions (Figure 2A). In order to test the effects of experimental condition and stimulus visibility, for each response type we conducted a separate 2 (experimental condition: ascending vs. descending)×7 (*g* value) mixed ANOVA, with response percentage as the dependent variable. Detailed results for these analyses are presented in Table 1. The percentage of “Nothing” responses decreased gradually as visibility (*g*) increased (see Table 1 and Figure 2A, left) and this pattern was similar for the ascending vs. descending condition, as indicated by the absence of effects for experimental condition or the interaction of the two factors. Post-hoc contrasts revealed that there were significant drops in the percentage of “Nothing” responses from *g* = 0.05 to *g* = 0.20 (see Table 1 and Figure 2A, left).

**Table 1:** Analysis of variance results.

Dependent variable	Factor	*MSE*	*F*	*η_p_* ^2^	*g* change intervals†
% “Nothing”	EC	22.15	0.26	0.01	
	*g*	95411.29	208.92***	0.90	[0.05, 0.20]***
	EC×*g*	330.53	0.72	0.03	-
% “Uncertain”	EC	177.85	2.28	0.09	
	*g*	4355.42	8.11**	0.25	[0.05, 0.25]**
	EC×*g*	3258.11	6.06**	0.20	A: [0.05, 0.10]* [0.10, 0.30]*D: [0.25, 0.30]**
% “Seen”	EC	325.54	3.63	0.13	
	*g*	85160.39	443.67***	0.95	[0.00, 0.25]*
	EC×*g*	1921.88	10.01***	0.29	A: [0.05, 0.30]*D: [0.00, 0.20]* [0.25, 0.30]*
% Incorrect	EC	32.03	0.72	0.03	
	*g*	52749.14	191.65***	0.89	[0.05, 0.20]**
	EC×*g*	179.76	0.65	0.03	-
% Correct	EC	59.79	0.97	0.04	
	*g*	75232.01	143.25***	0.86	[0.05, 0.25]***
	EC×*g*	2921.43	5.56**	0.19	A: [0.05, 0.30]*D: [0.05, 0.20]** [0.25, 0.30]*
Fix. spread	EC	541.32	5.90*	0.37	
	*g*	2002.62	21.65***	0.68	[0.00, 0.10]**
	EC×*g*	685.14	7.41***	0.43	A: [0.00, 0.10]*D: -
Norm. fix. count	EC	0.00			
	*g*	4.59	11.52***	0.54	[0.10, 0.15]*
	EC×*g*	1.38	3.46*	0.26	A: [0.15, 0.25]*D: [0.10, 0.15]** [0.25, 0.30]*
Avg. fix. duration	EC	8586.72	1.19	0.11	
	*g*	39879.94	4.34*	0.30	[0.15, 0.20]*
	EC×*g*	3987.81	0.43	0.04	-
Local contour density	EC	2.76	0.89	0.08	
	*g*	289.96	63.68***	0.86	[0.00, 0.15]**
	EC×*g*	12.72	2.79*	0.22	A: [0.05, 0.15]**D: [0.00, 0.10]**
Dot displacement	EC	4205.17	1.41	0.12	
	*g*	253849.30	39.32***	0.80	[0.00, 0.10]*** [0.15, 0.20]** [0.25, 0.30]**
	EC×*g*	51823.92	8.03***	0.45	A: [0.00, 0.10]**D: [0.00, 0.10]* [0.15, 0.30]*

*Note:* EC = experimental condition (Ascending vs. Descending); A = Ascending; D = Descending. ANOVAs were conducted with Huynh-Feldt correction.

† Post-hoc repeated contrasts.

* *p*<0.05;

** *p*<0.01;

*** *p*<0.001.

The value of *g* had a significant effect on the percentage of “Uncertain” responses, but this effect manifested differently in the two experimental conditions (as indicated by a significant g× experimental condition interaction; see Table 1). More precisely, in the ascending condition there was a clear peak at *g* = 0.10, reaching 46.46% (post-hoc contrasts indicated significant increases from *g* = 0.05 to *g* = 0.10, and decreases from *g* = 0.10 to *g* = 0.30; see Table 1 for details). In the descending condition, subjects reported uncertainty in less than 22% of cases, and this was, on average, relatively constant across different visibility levels (the trend was flat for *g*s between 0 and 0.25 and had a significant increase only from *g* = 0.25 to *g* = 0.30) (Figure 2A, middle).

“Seen” responses increased with increasing *g* value from *g* = 0 to *g* = 0.25, according to a sigmoidal curve (Figure 2A, right). The effect of *g* interacted with experimental condition: there was a steady increase in response percentage from *g* = 0.05 to *g* = 0.30 in the ascending condition, and from *g* = 0 to *g* = 0.20 in the descending condition, where we also found a drop in response percentage from *g* = 0.25 to *g* = 0.30. There was no effect of experimental condition alone on “Seen” response percentage.

We next carried out the same type of analysis−2 (experimental condition)×7 (*g* value) mixed ANOVA (see Table 1 for results details)−grouping the data according to verbal response accuracy (percent of correct and incorrect responses) (Figure 2B). Results were, with very few exceptions, similar to those found when grouping data according to response type. In the first block (*g* = 0) subjects reported correctly that there was “Nothing”, but their failure to detect objects for larger visibility levels (e.g., *g* = 0.05) led to a peak of incorrect responses (Figure 2B left) that was then progressively reduced with increasing *g*. The percentage of incorrect responses (Figure 2B, left) was affected by *g* (after the initial peak at *g* = 0.05, there was a steady drop until *g* = 0.20), but not by experimental condition or the interaction of the two variables.

Correct responses were a majority in the first block (response “Nothing” to lattices containing no object-related information when *g* = 0), but then dropped for *g* = 0.05, and subsequently increased across a smooth sigmoidal threshold (Figure 2B, right). As in the case of “Seen” responses, there were significant effects of *g* (a steady increase from *g* = 0.05 to *g* = 0.25) and experimental condition×*g* value (ascending: a performance improvement from *g* = 0.05 to *g* = 0.30; descending: improvement from *g* = 0.05 to *g* = 0.20, with a performance drop between *g* = 0.25 and *g* = 0.30). However, overall correct recognition performance did not differ between the two groups of subjects (i.e., no effect of experimental condition alone).

Grouping of data according to response type (“Nothing”, “Uncertain”, “Seen”) reflects the dependence of *subjective* recognition on *g*. When responses are grouped according to correctness of verbal response (incorrect, “Uncertain”, correct), this represents a more *objective* dependence of recognition performance as a function of *g*. To quantify recognition thresholds, we fitted subjective (Figure 2A, right) and objective (Figure 2B, right) recognition curves (portion with *g*>0) with a sigmoid function, for each subject (see Materials and Methods, Sigmoid fitting for threshold identification). In the case of subjective curves (Figure 2A, right), recognition thresholds were significantly lower for the descending (*ϕ_descending_* = 0.09) than for the ascending (*ϕ_ascending_* = 0.12) condition [*t*(24) = 4.41, *p*<0.001, *d* = 1.80] (Figure 2C, left). The same was true for the objective recognition curves (Figure 2B, right), with thresholds significantly lower for the descending (*ϕ_descending_* = 0.08) than for the ascending (*ϕ_ascending_* = 0.13) condition [*t*(24) = 4.95, *p*<0.001, *d* = 2.02] (Figure 2C, right). Within each experimental condition, the subjective and objective recognition thresholds were not significantly different (paired-samples *t*-test: *t*(12) = 2.11, *p* = 0.06 for the ascending and *t*(12) = 0.69, *p* = 0.50 for the descending condition) and were highly correlated (*r* = 0.94, *p*<0.001 for the ascending and *r* = 0.87, *p*<0.001 for the descending condition).

**Figure 3:**
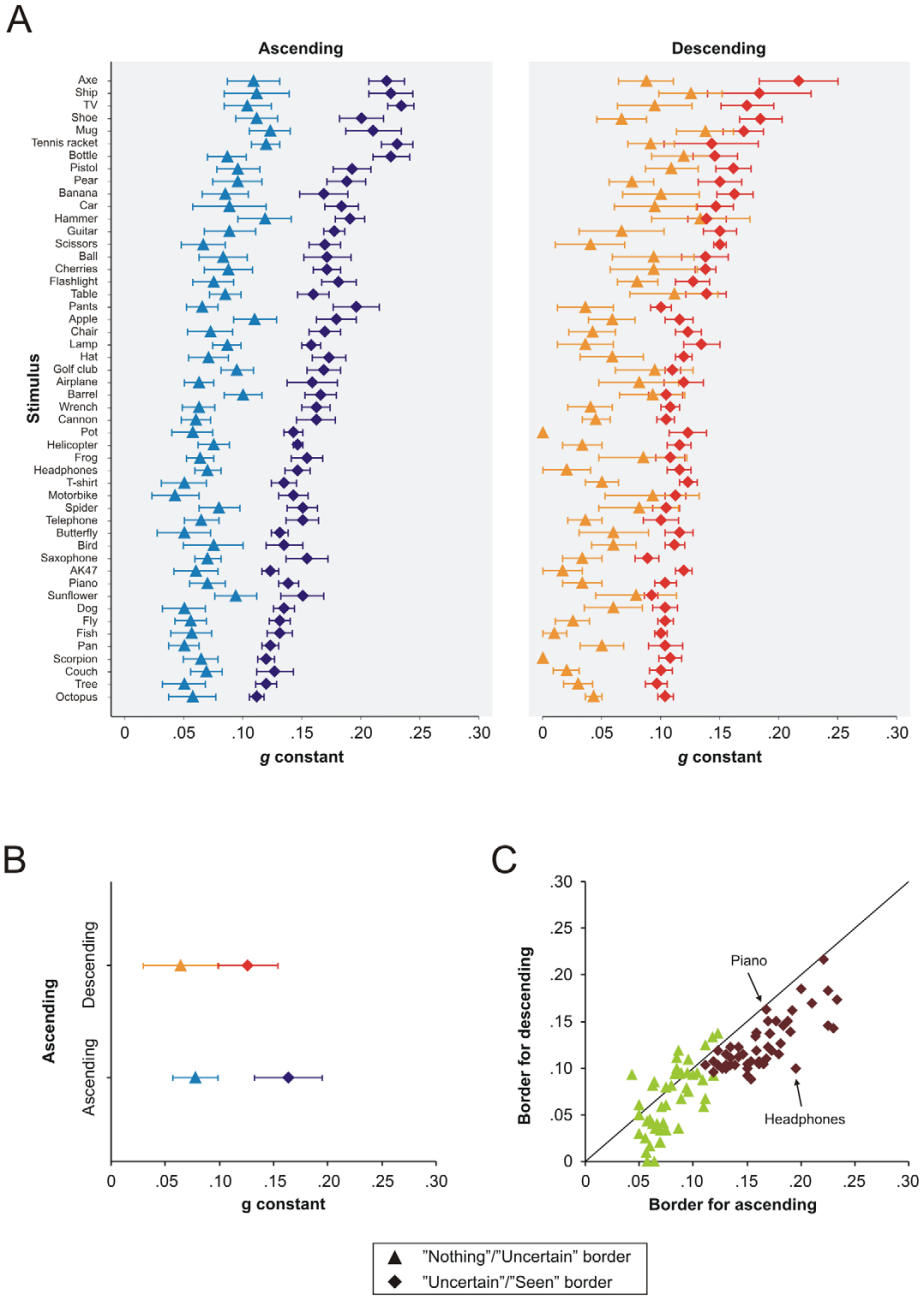
Detection and recognition of individual objects. (A) Borders between “Nothing”/“Uncertain” (detection) and “Uncertain”/“Seen” (recognition) computed on individual objects, for the ascending (left) and descending (right) conditions. Error bars are s.e.m. (B) Average and SD of detection and recognition borders from (A) across all objects. (C) Individual object detection and recognition borders in the descending versus ascending condition.

The above recognition thresholds were computed for each subject by taking performance curves across the entire set of objects. We next turned to investigate how individual objects were detected and recognized. To this end, for each object we computed the separation border between “Nothing”/“Uncertain” (detection) and between “Uncertain”/“Seen” (recognition), by finding the lowest *g* value where “Uncertain” and “Seen” responses occur, respectively. Results revealed that separation borders were specific to individual objects (Figure 3A). The two borders were also correlated and this correlation was higher for the ascending (*r* = 0.79, *p*<0.001) than for the descending condition (*r* = 0.58, *p*<0.001). This indicates that detection and recognition borders were more coherently modulated by object identity for the ascending than for the descending condition. In other words, if the detection border was lower/higher, then the recognition border was also lower/higher for the same object, but this relation was more prominent for the ascending condition. On average across objects, borders in the descending condition were both lower than their corresponding borders in the ascending condition (Figure 3B; *t*(49) = 3.74, *p*<0.001 and *t*(49) = 12.19, *p*<0.001 for detection and recognition, respectively; paired-samples t-test). This was the case for individual objects as well. The detection border was, for most objects, lower in the descending than ascending condition, and this effect was even clearer for the recognition border, where it held for all objects (Figure 3C). In addition, the magnitude of the border-lowering effect by experimental condition was object-specific because the effect was stronger for some objects (e.g., “headphones”) than for others (e.g., “piano”) (see Figure 3C).

**Figure 4:**
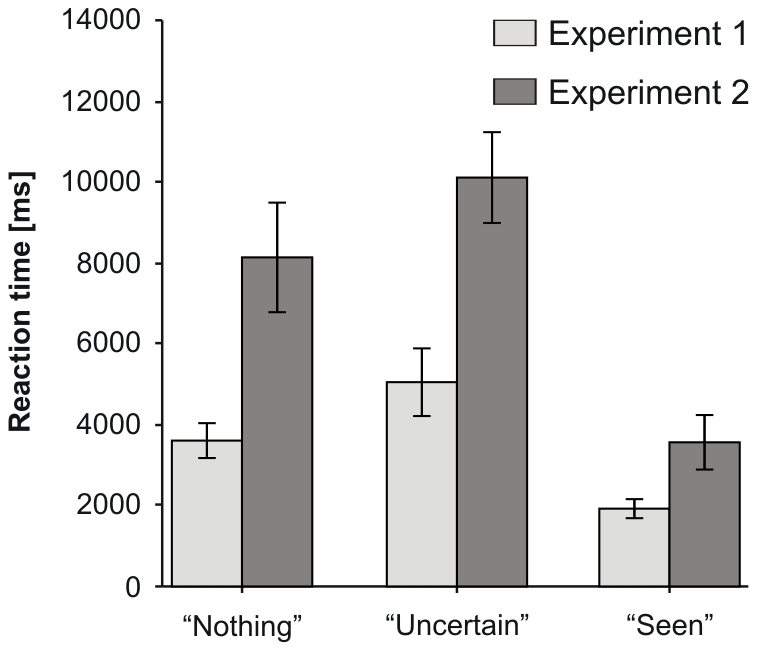
Exploration time estimated by measuring reaction time as a function of response type in the two experiments. Error bars represent s.e.m.

Lastly, we investigated RT differences between response types and as a function of experiment (Figure 4). For each subject, we extracted median RTs for each *g* value and then averaged them for each response type to obtain a global RT. RTs differed significantly between subjects included in Experiment 1 versus Experiment 2. We therefore included experiment as a separate independent variable in this analysis. We conducted a 2 (experiment: 1 vs. 2)×3 (response type: “Nothing”, “Uncertain”, “Seen”) mixed ANOVA with RT as the dependent variable. Overall, RTs were faster for subjects in Experiment 1 compared to those in Experiment 2: *F*(1, 24) = 13.54, *p*<0.01, *η_p_* ^2^ = 0.36. We also found a significant effect of response type: *F*(2, 48) = 47.09, *p*<0.001, *η_p_* ^2^ = 0.66; post-hoc pairwise comparisons with Bonferroni correction indicated that subjects were fastest when responding “Seen” and slowest when responding “Uncertain” (all comparisons were significant at *p*<0.01). Finally, we found a significant experiment×response type interaction [*F*(2, 48) = 6.63, *p*<0.01, *η_p_* ^2^ = 0.22], explained by the fact that the effect of response type was smaller for Experiment 1 (*η_p_* ^2^ = 0.53) compared to Experiment 2 (*η_p_* ^2^ = 0.73) (see Figure 4 for details). These results show that instructions to subjects related to RT did have effects on visual exploration. However, the relation between trial durations across different response types remained the same, i.e. lack of instruction regarding response speed merely scaled up RTs.

### Eye tracking data

To shed new light on the integration of visual information during free visual exploration and on how visual exploration is affected by previous exposure, we complemented the psychophysical measurements with eye-tracking recordings. We investigated how eye movement patterns differed depending on the availability of sensory evidence and previous exposure (top-down processes). To this end, we monitored eye position for subjects in Experiment 2 and computed the patterns of saccades/fixations (see Materials and Methods, Identification of fixations). The analyses conducted on this data were similar to the ones conducted for response percentages – i.e., 2 (experimental condition: ascending vs. descending)×7 (*g* value) mixed ANOVAs, with fixation-related measures (see below) as the dependent variable in each case (see Table 1 for details on statistical results).

**Figure 5:**
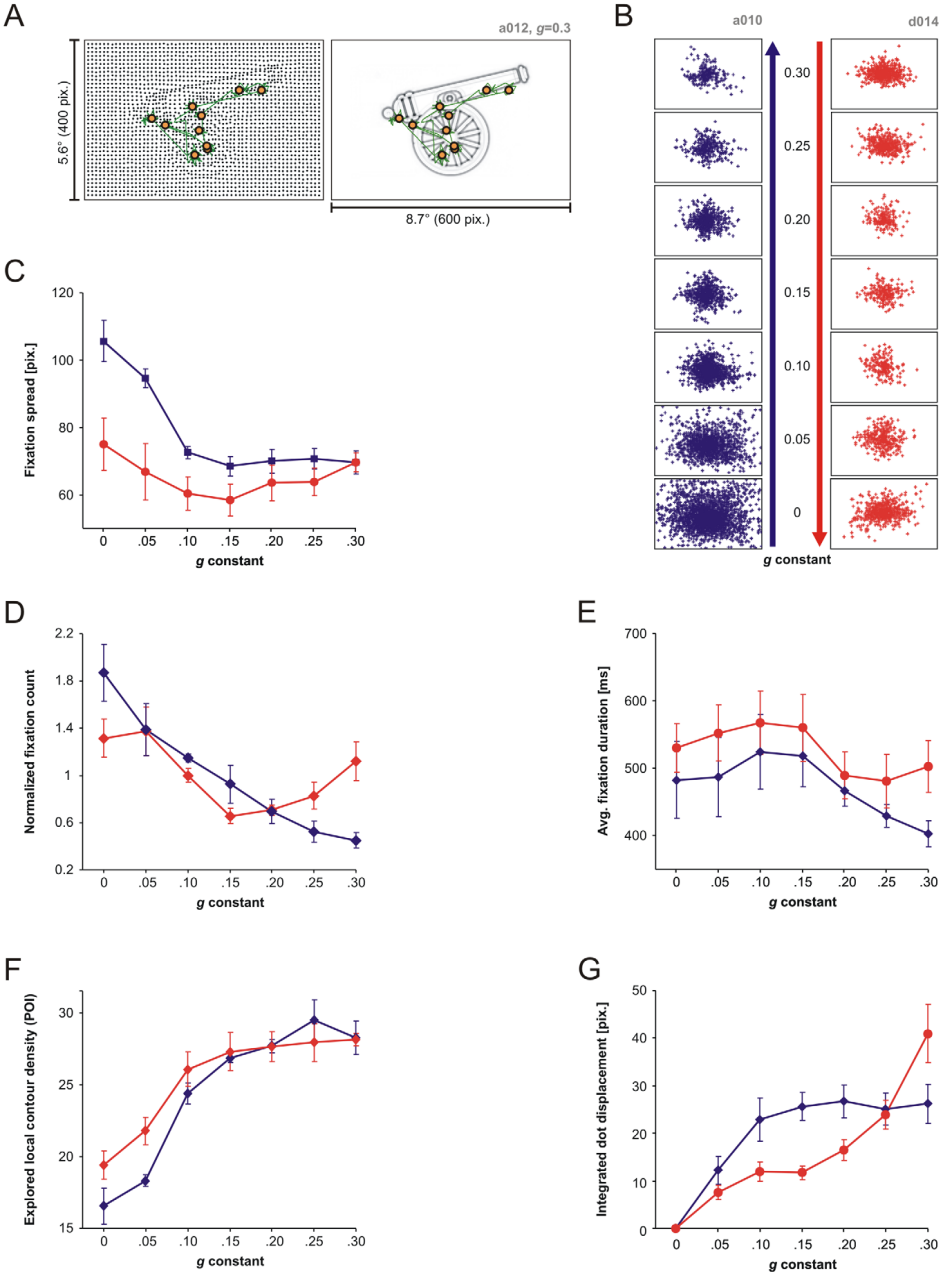
Eye-tracking analyses. (A) Pattern of fixations/saccades revealed in relation to the “cannon” stimulus (left) and its underlying POI map (right). (B) Pooled fixations on all stimuli as a function of visibility for a subject performing the ascending (left) and one performing the descending protocol (right). (C) Average fixation spread (distance from image center). (D) Fixation count, normalized per subject. (E) Fixation duration. (F) Average local contour density (computed from POI map) in areas of 0.5° in diameter around explored locations corresponding to fixations on the dot stimulus. (G) Integrated dot displacement, computed as a sum of displacements of dots (in areas of 0.5° in diameter around each fixation) relative to the undeformed lattice. The sum runs over all fixation locations in the trial. Error bars represent s.e.m.

When the stimulus was visible, fixations were localized in stimulus areas where dot displacement (Figure 5A, left) represented the underlying local contour density from the POI map (Figure 5A, right). The spatial distribution of fixations was markedly different in the ascending versus the descending condition. Fixations for all objects are shown for two representative subjects (one performing the ascending the other the descending task) across different *g* values in Figure 5B. In the ascending condition subject *a010* explored almost the entire stimulus surface at *g* = 0. As visibility was increased, fixations became more concentrated towards the center, where objects were located (Figure 5B, left). This was not the case for a subject (*d014*) that viewed stimuli in descending order of visibility. In this case, the spatial extent of fixations at *g* = 0.3 was compressed for intermediate visibility levels and then expanded again as *g* approached 0 (Figure 5B, right). The reported effects were also consistent when we investigated population data and computed the distances of fixations from the center of the stimulus (fixation spread) (Figure 5C). Overall, fixation spread was significantly larger in the ascending versus descending condition. We also found a main effect of *g* (see Table 1 for details), but the *g*-related curves differed, as indicated by a significant experimental condition×*g* interaction. Fixation spread was largest in the ascending condition for *g* = 0 and then decreased until *g* = 0.10. For the descending condition, fixation spread had a “U” shape [quadratic trend: *F*(1, 5) = 7.66, *p*<0.05, *η_p_* ^2^ = 0.60].

To further describe the image exploration process we next investigated fixation count per trial and fixation duration. For each subject, fixation count was first normalized to the individual average because its absolute value tended to be highly subject-specific (fixation count per trial is tightly related to reaction time, and thus to individual subjects' exploration strategy). Normalized fixation count was not affected by experimental condition, but it changed as a function of *g* (see Table 1) and the interaction of *g* with experimental condition: it decreased monotonically and linearly in the ascending condition [linear trend: *F*(1, 5) = 19.77, *p*<0.01, *η_p_* ^2^ = 0.80] and was “U”-shaped in the descending condition [quadratic trend: *F*(1, 5) = 129.56, *p*<0.001, *η_p_* ^2^ = 0.96] (Figure 5D). In addition, at maximum visibility (*g* = 0.3) subjects made significantly more fixations in the descending than ascending condition [*t*(10) = 3.75, *p*<0.01; independent-samples t-test], indicating that stimulus novelty was a factor influencing fixation count. Fixation duration was modulated differently by visibility as it varied only as a function of *g*, while experimental condition or its interaction with *g* had no statistically significant effect. In both conditions, the duration of fixations was longer for low and intermediate visibility levels (significant decrease from *g* = 0.15 to *g* = 0.20; see Figure 5E) indicating that subjects tended to maximize the integration of available visual features under difficult viewing conditions. In addition, stimulus novelty also played a role because at maximum visibility (*g* = 0.3, which is the first block in the descending protocol) the descending (versus ascending) condition was associated with significantly longer fixations: *t*(10) = 2.33, *p*<0.05.

Local contour from the original image is a hidden variable because subjects have only indirect and limited access to it by means of dot displacement. It is however possible to estimate how subjects accessed this information. To this end, we considered locations of fixations on the stimulus (dot image) and then computed the corresponding underlying average local contour density (from the POI map) in a region spanning 0.5° visual angle around each fixation. We found that the average local contour density in explored locations increased with increasing *g* value, indicating that information about local contours was progressively made available to subjects and they used this information to reach a decision (Figure 5F): we found no difference between the two experimental conditions, but there was an effect of *g* (increase from *g* = 0 to *g* = 0.15) and of the experimental condition×*g* interaction (increase from *g* = 0.05 to *g* = 0.15 in the ascending condition, and from *g* = 0 to *g* = 0.10 in the descending condition). These analyses reveal that explored contour density reached a plateau around *g* = 0.10–0.15, showing that already around the observed recognition thresholds subjects were able to seek out most informative locations. In addition, at very low visibility levels subjects in the descending condition were able to more efficiently explore informative locations than in the ascending condition [at *g* = 0.05: *t*(10) = 3.25, *p*<0.01; independent-samples t-test], suggesting that top-down effects can guide visual exploration (Figure 5F).

Finally, we investigated how subjects used the information available in the stimulus itself, that is, the deformation of the lattice by displacing the location of dots. We considered each fixation and computed the displacement of dots from the grid (contribution of jitter not included), within an area of 0.5° around the fixation. We then computed the total integrated dot displacement per trial by summing displacements over trial fixations. Intuitively, this measure reflects the amount of dot displacement integrated by the subject to reach a decision. Results indicate that integrated dot displacement (Figure 5G) varied as a function of *g* (increases from *g* = 0 to *g* = 0.10, from *g* = 0.15 to *g* = 0.20 and from *g* = 0.25 to *g* = 0.30) and experimental condition×*g* (an increase from *g* = 0 to *g* = 0.10 in the ascending condition, and an increase from *g* = 0 to *g* = 0.10 and *g* = 0.15 to *g* = 0.30 in the descending condition), but not experimental condition alone. Thus, in the ascending condition integrated dot displacement saturated already around the perceptual threshold. Even though displacement increased with increasing *g*, subjects explored roughly the same amount of dot displacement to reach a decision by making progressively fewer fixations (see Figure 5D). In the descending condition, subjects first explored a large amount of displacement at maximum visibility (g = 0.3), because of stimulus novelty, but at intermediate visibility levels they integrated less dot displacement than did subjects in the ascending condition [independent samples t-test: t(10) = 4.25, p<0.01 for g = 0.15; t(10) = 2.50, p<0.05 for g = 0.20]. The latter effect suggests that strong priming can have a dramatic influence on the integration of visual information required to support a decision.

## Discussion

During natural vision, humans actively explore their visual environment and are able to map known patterns onto perceptually defined categories. Visual exploration relies on processes such as saccades and fixations [27] and supports an amazingly robust mapping between visual patterns and object categories during recognition. To effortlessly classify objects, humans can use most available information extractable from visual features, such as contour, color, depth, motion, texture, shading etc [28]. For this reason, under natural viewing conditions recognition is highly robust and occurs almost instantaneously. The investigation of object recognition thus requires a strategy to enable the manipulation of object perception in a controlled fashion.

One of the most popular approaches in the study of object perception is masking, which limits the exposure duration of the stimulus (e.g., see [18], [29]). We argue that this prevents the possibility to investigate active visual exploration and may hinder the study of realistic, natural vision, for several reasons. First, transients due to brief presentations of visual stimuli induce onset and offset responses of visual cortex neurons [30]–[32]. Such responses may have confounding effects on the study of object perception because one cannot dissociate integration of visual information from other cortical mechanisms related to temporal dynamics of the neuronal populations' activity. Second, the use of masks to limit subjects' access to visual information is controversial [33] because the mask may not effectively remove object-related information. The latter may persist for hundreds of milliseconds in visual cortex, even following a mask [34]. Other methods to study object recognition can be employed without necessarily flashing stimuli and hence without masking, being useful to reveal different aspects related to object perception, such as visual exploration. These methods include transformative [1]–[5], [7], [8], [10] and generative techniques [13]–[16] that both offer control over the perceptual process by manipulating object-related information content in the stimulus itself rather than by restricting visual access to it.

The most powerful transformative methods, such as RISE [7], [9]–[11], [29] or SNR manipulation [8], can preserve global image statistics, such as global luminance, contrast, or spatial frequency and enable the control of object perception across a continuous domain. In addition, transformative techniques include a large amount of visual features corresponding to the object because they manipulate the original image itself. Thus, they have the advantage that one can also devise visual tasks involving fine object discrimination, i.e. subordinate-level categorization (e.g., telling apart a dog breed from another). Among disadvantages, we first note that although global image statistics can be preserved, the transformations involved usually do not preserve local image properties (e.g., local luminance). Furthermore, in transformative techniques one has little control over the image features that are affected by the transformation because multiple features are present in the stimulus (contour, shading, texture, possibly color, local luminance, etc). As an example, one cannot precisely control how object contour is affected during image degradation or phase randomization. Improvements and workarounds are of course possible, but by their nature transformative techniques do not isolate individual features, and thus, their controlled expression in the stimulus is more difficult.

Generative techniques solve some of the problems of transformative methods by isolating a limited set of visual features corresponding to the object, a process in which many visual details are lost. Isolated features (e.g., contour) are included in a controlled fashion in the generated stimulus by using local elements, such as oriented Gabors [13]–[15] or oriented line segments [35]. Generative methods have the advantage that features available to the subject for visual recognition can be precisely controlled. Furthermore, the manipulation of these features via local elements (e.g. by changing the orientation of local Gabor patches or line segments) keeps image statistics (luminance, contrast) relatively constant, both globally and locally. However, by their nature, generative techniques have the major pitfall that they cannot convey detailed visual information about the object and hence cannot be used for subordinate-level categorization. In this respect, generative and transformative techniques should be considered complementary.

The method we presented here is a novel generative technique. Many previous methods in this category render a set of local elements in fixed spatial locations and encode a relevant visual feature by changing the properties of these local elements (e.g., orientation of Gabors [13]–[16]). By contrast, our method uses homogeneous local elements (dots – but other self-symmetric elements are also an option), which by themselves carry no relevant visual information, and manipulates their position. Local contour information from the original image is encoded into the stimulus by deforming the lattice onto which the local, identical elements are rendered. By progressively deforming the regular lattice of dots, the method allows to manipulate the amount of object-related information conveyed to the subject. Lattice deformation effectively transfers local contour information corresponding to the object into the stimulus, but many features from the original image, such as color, texture, luminance, and so on, are missing. The progressive deformation of the dot lattice, via a single parameter, creates novel stimuli that are progressively more homologous to the original image of the object in terms of local information. In addition, lattice deformation keeps global image statistics such as luminance and contrast constant, and, at visibility levels where subjects already perceive objects, local image statistics are affected very little. For example, at threshold visibility (*g*∼0.10–0.20) the lattice is minimally deformed, with average dot displacement as low as 1.57 pixels (*SD* = 0.13 pixels) representing 0.023° visual angle (stimulus gallery available online – see Materials and Methods, Stimuli).

The present stimulus-generation technique was developed mainly to enable investigation of visual inference under conditions with limited object-related information [36], [37]. As such, the method allows subjects to incrementally integrate visual evidence. The setup presented here is not a typical visual search but rather a visual exploration paradigm [38] because the local elements are all identical (dots) and there are no distractors in the classical sense [39]. By contrast, in methods relying on contour integration the same property of local elements (Gabor orientation) is usually manipulated for both elements that represent the target (the contour) and for elements that do not contain object-related information. The latter elements act as distractors [13]–[15] in a similar fashion as in visual search tasks [39]. We tried to separate visual search from visual integration in the present paradigm. That this is indeed achieved is demonstrated by fact that in the “Uncertain” condition (having longest exploration time) the patterns of fixation are restricted almost entirely to the location of the object. Indeed, subjects detect the object but they cannot recognize it. This happens not because distractors impair the detection process (the location of relevant visual information is clear to the subject) but because visual information is too limited to reach a perceptual decision. Thus, in the “Dots” method recognition difficulty is manipulated by controlling the amount of information available for visual integration and not by using distractors.

We used a single parameter to manipulate the amount of object-related information in the stimulus, i.e. *g* value, an advantage also shared by other methods [7], [8], [13]. The change of this single parameter produces significant changes in perception, leading to higher recognition levels as *g* increases. In statistical tests, we have found that this parameter was consistently effective on dependent variables, even at the level of visual exploration behavior, such as fixation pattern. The transition from not seen/not detectable to seen/identifiable stimuli is smooth across a set of objects and passes through a state of uncertainty where objects can be detected but not recognized.

As the dot lattice is progressively deformed, perception assumes all stages, from no detection, to detection without identification, and finally to successful identification. Thus, detection and identification do not occur instantaneously and simultaneously, as has been suggested for other stimuli [18], but seem to be separate processes, in line with recent findings [16], [19], [29]. Object detection under free visual exploration, as shown here, is not a trivial process. In our setup, detection not accompanied by recognition (“Uncertain”) determines subjects to explore for significantly longer durations than in cases where nothing is perceived or when the object can be clearly identified. This timing relation is preserved both when subjects are instructed that reaction time is a relevant variable and when this instruction is missing, but in the latter case reaction times are scaled up and are more variable. Although experiments reported here should not be considered reaction-time relevant in the classical sense, measurement of trial duration shows how visual exploration duration is correlated to perceptual outcome and demonstrates that uncertainty is always associated with longest exploration. It is therefore interesting to use the present method to investigate how the brain explores visual hypotheses [36], [40] and, by employing electrophysiological or fMRI techniques, to identify cortical processes related to visual exploration. Another interesting result is that as soon as subjects report that they can clearly see an object (beyond detection), they can also correctly identify it. Subjective (cases where subjects report to have clearly seen an object) and objective (cases where subjects report correctly the identity of the object) thresholds are identical.

Stimuli consisting of dots have been employed before to study visual perception [21], [41]–[48]. Uttal and colleagues have used dots to represent shapes such as alphabetic characters [47], lines, curves, and angles [41], and three-dimensional surfaces [42], embedded in a set of distractor, “masking” dots located randomly in space and time [41]. While parallels may be drawn to stimuli proposed here, there are several major differences. First, our dot stimuli can be generated automatically from original source images and can represent fairly complex objects whose depiction would be difficult if they had to be generated manually, as was the case with Uttal's dot shapes. Second, stimuli proposed here are static, their structure remaining fixed throughout stimulus presentation. Finally, instead of representing the object's structure quite clearly and using distractors to prevent immediate recognition [41], we distort a regular lattice that is not a distractor field but can be considered more like a reference field. Distortion of the dot lattice then manipulates perceptual processes underlying Gestalt phenomena such as good continuation and grouping. Dot lattices were extensively used to study Gestalt principles such as grouping by proximity or similarity. For example, Kubovy defined several types of dot lattices (e.g., hexagonal, rhombic, rectangular, square) [21] and showed that their geometry can be changed to flexibly manipulate grouping [43]. Here, we used dot lattices as a deformable regular reference in order to study object perception rather than basic Gestalt phenomena – but low-level principles of good continuation and grouping are most certainly involved in perception of dot stimuli introduced here.

In relation to the above-mentioned Gestalt phenomena, precise quantitative studies have been performed relying exactly on dot stimuli. Notable is the work of Feldman [44]–[46] who has studied in great detail how groups of dots are perceived as a function of perceptual task. Using groups of 3 [44], 4 [45], 5 or 6 dots [46], arranged in parametrizable configurations, he has shown that perceptual classifications of such configurations are consistent with a Bayesian model of contour integration. Thus, humans rely on Bayesian-like inference [44], [46], having prior probabilities imprinted by experience, when judging contour (and likely also shape [49]) information. These findings are especially relevant in the context of our stimuli because it is likely that subjects extract at least two types of visual features from dot stimuli: they integrate contours (Gestalt principle of good continuation) and evaluate dot densities (Gestalt principle of grouping by proximity). In regions where the local information in the original image is coherent along continuous contours, the POI map will contain preferentially responses from Gabors aligned to the local contour, thereby creating a coherent local displacement of dots along the respective contour. Such dot contours can be integrated by subjects when extracting information about the identity of the object. Understanding the underlying process of this integration is important [44]–[46], [50] and may be helpful in revealing how the fixation-by-fixation visual sampling process is guided. For example, it is now known that the curvature of the contour contains a large amount of information [51], [52] and that areas with concave contours are especially important when processing the boundaries of objects [52]–[54]. One may then use the proposed dot stimuli to investigate further how contours are integrated: Do subjects sample mainly regions with high curvature and among those are the concave contours explored preferentially? These questions can be addressed with the proposed stimuli because recognition is not immediate but there is substantial exploration relying on several fixations. In addition, dot contours in these stimuli follow contours with natural statistics, from images of real objects, therefore allowing one to study contour integration in a naturalistic, less constrained setting.

In addition to contours, local dot densities may also be used by subjects by virtue of grouping by proximity. Perception of dot densities has also been studied recently [55], [56]. Segmentation of dot densities into different parts was shown to depend on the distance between densities and the density of dot clouds [55]. In dot stimuli presented here, local dot density is proportional to the local energy extracted by Gabors in the POI map and therefore both the location and density of the clouds is modulated by the structure of the object. Perhaps a dot density segmentation is operational in guiding fixations in our stimuli, subjects exploring sequentially areas with high dot density (high POI energy). If such segmentation takes place, then it is interesting to study how segmented subparts are bound together during recognition. This may be a gradual process, such that during the uncertain state one is able only to segment local information but is unable to bind it. For object identification binding of the locally identified features into a coherent whole may be further required. Thus, one may use the proposed stimuli to investigate both segmentation and binding. It is possible that Bayesian-like inference observed by Feldman [44], [46] at the processing of low-level features, such as contour, may also be operational at the holistic level when binding locally identified dot densities during recognition. Finally, at this point, it is unclear if contours and dot densities contribute differently to recognition. Further studies relying on the proposed stimuli could elucidate how low-level processes such as contour integration and grouping drive accumulation of evidence to subserve object identification.

Object recognition is thought to involve both a fast, feed-forward sweep of coarse visual information [57], [58] and a feedback component that guides further detailed visual exploration [23], [59], [60]. Deformed dot lattices provide only coarse, low spatial frequency visual information about objects. As a result, visual exploration becomes more extensive because fine image structure to quickly guide exploration is missing. By presenting stimuli in blocks ordered according to ascending or descending visibility, we were able to touch on the non-trivial interaction between feed-forward and feedback processes. We found that the descending condition (where subjects first perceive the objects clearly) significantly lowers recognition thresholds as compared to the ascending condition, a phenomenon known as perceptual hysteresis [5]. Hysteresis is believed to be caused by feedback, which enables the perceptual system to identify an object that was recently seen, even when only little feed-forward information is available [17].

We found that visual hysteresis is manifested to different extent for individual objects, consistent with previous reports [7], [10] and it affects both detection and recognition. These results suggest that top-down modulations act to guide visual exploration, facilitating both recognition and detection, such that objects can be recognized and detected under poorer visibility conditions. In addition, borders for detection and recognition are object specific and are better correlated when subjects have no previous exposure to the set of objects (ascending condition) than when subjects have been previously primed with the easily identifiable stimuli (descending condition). This effect is likely produced by the change in exploration strategy induced by top-down processes in the descending as compared to the, naive, ascending condition. In the latter case, objects were explored more extensively, systematically, and those that were more difficult to detect were also more difficult to recognize, leading to a correlation between detection and recognition borders. By contrast, in the descending condition, subjects could recognize objects at lower visibility levels, even relying on subparts of objects to identify them, and this compressed the distance between recognition and detection borders. In addition, “Uncertain” response percentage did not exhibit a peak at intermediate visibility levels, as was the case in the ascending condition, but uncertainty remained, on average, at a baseline level. As a result, the detection border was more variable and more fuzzy in the descending condition because subjects frequently jumped from “Seen” directly to “Nothing” responses (without the uncertain state). This fuzziness of detection threshold also contributed to a lower correlation of detection and recognition borders. Thus, robust top-down knowledge lowers detection and recognition borders of individual objects, changes exploration strategy and perceptual decisions, and leads to a more polarized response pattern including more frequent “Seen” but less frequent “Uncertain” responses.

Eye-tracking analyses confirmed the above scenario. Subjects explored stimuli across a larger spatial extent in the ascending than the descending condition. In the latter case, top-down influences can decrease the spatial extent of exploration and the number of fixations even though the available visual information decreases (top-down knowledge compensates external information loss). This holds up to a point where feedback is not sufficient to support a perceptual decision and therefore sampling of external information increases again (thus the characteristic “U” shape of fixation pattern statistics in the descending condition). The effect of top-down guidance of visual exploration was evidenced robustly when we investigated how subjects explored and integrated visual information. As *g* value was scaled up, local contour information (from the POI map), although not directly accessible to subjects, was more efficiently explored by corresponding fixations on dot stimuli. Importantly, at low visibility levels top-down control (descending condition) guided fixations to regions of the object that had more underlying information in the hidden POI map (even if this information was not revealed or was very difficult to detect in the dot stimulus), a behavior that was not shared by naive subjects performing the ascending protocol. At intermediate visibility levels, the latter integrated clearly more visual information (measured as integrated dot displacement) to reach a perceptual decision than subjects which have previously seen the objects clearly (descending condition). Evidence shown here, revealed by psychophysics and fixation analyses, suggests that top-down processes induced by previous exposure to target objects not only modulate perception but can also guide visual exploration and optimize the integration of visual information. In such cases, subjects make fixations where informative locations of the objects were supposed to be, even if these are no longer present in the stimulus. Priming is possible not only by presenting images of targets but also by using non-visual congruent cues, such as words related to the identity of the object [2], [7]. It would be interesting to study whether such non-visual priming could affect visual exploration behavior as well.

Our findings add to previous studies that have emphasized the importance of feedback [2], [60], [61] and have shown that processes as early as figure-ground segregation may rely on it [62], [63]. A recent fMRI study by Strother and colleagues [64] has pointed out that in the absence of strong bottom-up cues, as is also the case with stimuli presented here, figure-ground segregation is associated to longer persistence of activation for upright than inverted images of faces and animals. This suggests that for difficult to perceive stimuli feedback from higher areas is actively involved as early as the primary visual cortices [64].

We also found that novelty (first exposure to stimuli in the descending condition) and low discriminability (small *g* values) were associated with an increase in fixation duration. The latter was reported to be unrelated to stimulus familiarity but more likely connected to cognitive demand [65], [66]. This is also suggested by increased fixation duration when subjects identify new over old stimuli [67]. Because stimulus parameters and task load are under control, the present method allows tackling this problem more precisely. Our results confirm that increased fixation duration is due to increased cognitive demand (novel type of stimulus/unknown objects in the first block of descending condition and low discriminability for low *g* values). This further indicates that under conditions with high cognitive demand the brain optimizes sampling of visual information not only by guiding fixation location during visual exploration but also by controlling the amount of integration per fixation.

The use of deformed dot lattices to represent objects is not without drawbacks. First, high spatial frequency details of objects are lost. Therefore, it is not possible to use the method to dissociate between objects that are part of the same category and have very similar shapes (subordinate level categorization). Fine discrimination, between e.g. two similar dog breeds, is often impossible with such stimuli. For subordinate-level categorization transformative methods such as RISE [7] may be more appropriate. Second, because visual information is limited to the approximation of local contours, the method requires that the object is first isolated from background before computing the POI map. Background is not distinguishable from the object in deformed dot lattices and may heavily interfere with detection and identification. Finally, humans are very good at detecting regularity, such that, for a perfect lattice of dots, they can immediately realize that no object is there and therefore quickly cease to explore the stimulus. To compensate for this problem addition of noise to the lattice is required. This prevents subjects from directly telling that no object is present and motivates them to actively explore the stimulus.

To conclude, the “Dots” stimulus-generation method is useful for investigating object recognition under free visual exploration. The precise control over visual information and the ability to relate it to quantitative properties of the stimulus could open the way for a new generation of studies investigating object recognition and free visual exploration.

## Materials and Methods

### Ethics Statement

Psychophysical and eye-tracking measurements were performed on human subjects who gave their prior written informed consent to participate in the experiments. The experimental protocols have been approved by the local ethics committee of the University of Medicine and Pharmacy “Iuliu Haţieganu” of Cluj-Napoca (approval No. 150/10.12.2009).

### Stimuli

The original images used for generating the stimuli were selected from the Caltech 101 [68], Caltech 256 [69] and ETH-80 [70] databases, or from various internet sources. We used 50 images (plus 2 additional images for the practice trials) of different plants, fruits, animals and human-made objects (Figure 1D). Images were rescaled such that the image frame occupied the same size (600×400 pixels) and, additionally, all background information was removed and the object of interest was centered in the frame. The lattice deformation procedure was applied to each image, using a lattice of black dots on a white background (600×400 pixels) with a dot diameter of 5 pixels and a distance between dots of 10 pixels. The elastic constant *K* was set to 10, and the distance *h* between POI and lattice planes was set to 5. Seven stimuli were generated from each image, corresponding to seven different gravitational constant (*g*) levels, from 0 to 0.3, in steps of 0.05. To prevent subjects from instantaneously detecting that no object was present when *g* = 0 (perfect, undeformed lattice), we added a uniform random jitter of zero-mean and 3 pixels maximum amplitude to the position of dots after deformation. This small jitter was applied for all levels of visibility. Examples of dot stimuli generated from the same source image are presented in Figure 1C. Thus, the final stimulus set consisted of 350 stimuli and an additional 14 for the practice trials (the full set of stimuli is freely available under http://www.raulmuresan.ro/sources/lattdef).

Object recognition difficulty from dot stimuli depended to a large extent on the physical spacing between dots and on the distance of the subject from the monitor. Therefore, we had to calibrate monitor distance such that subjects had a very hard time identifying the objects for *g* = 0.05 and could effortlessly identify them for *g* = 0.3. After the calibration, stimulus images of 600×400 pixels spanned 8.7°×5.6° of visual angle corresponding to an inter-dot spacing of 0.1015° in the original undeformed lattice. Stimuli were displayed on a 22 inch Samsung SyncMaster 226BW LCD monitor with fast response time (2 ms), placed at a distance of 1.12 meters from the subject. Viewing distance was maintained using a chinrest.

### Experiments

Because the present study focused on free visual exploration, the task was designed for measurement of accuracy rather than reaction time. Nevertheless, because we wanted to quantify the duration of exploration for different response types we additionally measured reaction times. All results regarding reaction times should therefore be interpreted not in the classical sense but as a reflection of exploration duration. Two different experiments were carried out. In **Experiment 1**, instructions given to subjects emphasized accuracy, but mentioned that speed was important as well (see Procedure for details). In **Experiment 2**, subjects were given no instructions regarding response speed. In addition, in the latter experiment we carried out concurrent eye-tracking to identify the pattern of saccades/fixations during each trial.

### Subjects

Twenty six subjects (15 females), aged 20–34 years (*M* age = 25.88, *SD* = 4.20), took part in the study. They were either volunteers or undergraduate psychology students who received course credit for participation. All had normal or corrected-to-normal vision, and no known neurological or visual impairments. Fourteen subjects (10 female, *M* age = 23.93, *SD* = 2.64) participated in the first experiment and twelve (5 female, *M* age = 28.17, *SD* = 4.61) in the second experiment. In each experiment, subjects were assigned to one of two experimental conditions (see Experimental Design), resulting in *N* = 7 and *N* = 6 subjects in each condition for Experiment 1 and 2, respectively.

### Procedure

Subjects were instructed that upon viewing each target stimulus their task was to decide whether the dot pattern represented something meaningful. They had to respond by pressing buttons “A” (if they decided that nothing meaningful was there—response “Nothing”), “S” (if they thought they saw something but were uncertain what it was—response “Uncertain”) or “L” (if they perceived something meaningful in the pattern and knew what it was—response “Seen”). Each trial started with a fixation mark, presented centrally for a random variable duration (500–1000 ms in Experiment 1 and 1500–2000 ms in Experiment 2). The fixation mark was followed by the target stimulus, presented continuously until the subject responded by pressing one of the three buttons. After the button-press response, a message was displayed on the screen asking subjects to verbalize their response. An experimenter was present in the room throughout the experiment and manually recorded the subject's verbal responses. When subjects were able to identify an object they had to name it explicitly. When they were uncertain about the object, they were instructed to guess, if they could. The task started with a practice block of 14 trials, followed by 7 experimental blocks, with a break period after each block. A block consisted of 50 trials corresponding to stimuli obtained for the 50 objects at a given *g* level. Within each block, presentation order of stimuli was randomized.

In Experiment 1, it was stressed that subjects should press one of the three response buttons as soon as they had reached a decision, but to take as much time as was needed to reach that decision. It was also suggested that accuracy was slightly more important than speed. In Experiment 2 subjects were only told to take as much time as was needed to respond, with no mention that reaction time was a relevant variable.

In Experiment 2, an ASL EyeStart 6000 system was used to record eye movements (see Identification of fixations). Calibration was conducted before each experimental block using a nine-point display, according to the indications included in the manufacturer's manual. To correct for potential shifts of eye position estimates in between calibrations, the fixation mark was presented for an extended duration compared to Experiment 1. Subjects were instructed to maintain precise fixation on the fixation mark at the beginning of the trial and this information was later used to correct for potential shifts in each trial.

### Experimental design

In both experiments we used a between-subjects design, with two versions of the task: (1) ascending (each block contained stimuli with the same *g* level – corresponding to the 50 objects in Figure 1D – and blocks were ordered from *g* = 0 to *g* = 0.3); (2) descending (this version was similar to the previous one, except that blocks were presented in reverse order of *g* value – i.e., from *g* = 0.3 to *g* = 0). We used these two conditions in order to investigate whether previous exposure affects stimulus visibility. Whereas in the ascending condition visibility relies mainly on stimulus evidence, in the descending procedure visibility relies on an interaction between top-down sensory expectations and stimulus evidence. Each subject was assigned to one of these two conditions.

### Sigmoid fitting for threshold identification

To identify thresholds of object recognition we fitted the sigmoidal-shaped response and accuracy curves corresponding to each subject with a sigmoidal function *f_sig_* dependent on the gravitational constant, *g*:
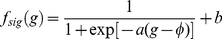
(12)where, *a* is the slope of the sigmoid at the threshold, *b* is the vertical offset, and *ϕ* is the horizontal shift on the *g* axis, i.e. the threshold.

The fit was implemented using a gradient descent method to minimize approximation error. Instead of using percentages, values of the accuracy curves were normalized to the interval [0..1] before fitting, to match the sigmoid function described in Eq. 12. The relevant parameter for our purposes was *ϕ*, that is, the threshold. It represents the point on the *g* axis where the sigmoid function crosses half of its maximum amplitude (offset not considered).

### Identification of fixations

Fixations were identified by a simplified version of the velocity based algorithm introduced by Nyström and Holmqvist [71] that uses two adaptive velocity thresholds. A saccade is detected when the velocity of eye movements rises above the saccade identification threshold, *v_I_*. A second, lower, threshold, *v_S_*, is used to identify the onset and ending of the detected saccade. The original algorithm [71] starts with a high *v_I_* and in each iteration updates its value according to the formula:
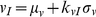
(13)where, *μ_v_* and *σ_v_* are the mean and standard deviation respectively of all velocities smaller than *v_I_*, and *k_vI_* = 6.

The iterative process stops when the difference between two successive values of *v_I_* is below 1°/s. Next, the saccade onset threshold, *v_S_*, is computed in similar fashion:
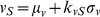
(14)where, *k_vS_* = 3.

In our case, setting *k_vI_* = 3 and *k_vS_* = 1.5 yielded better saccade identification. As a difference from the Nyström and Holmqvist algorithm [71], we could not identify glissades because our eye-tracker sampling period (20 ms) was in the range of glissade duration. Therefore, we used the same threshold to identify both saccade onset and ending. Once saccades have been identified, fixations were defined as samples between successive saccades. Fixations in which more than half of the eye position samples could not be correctly recorded by the eye-tracker (due to pupil loss, corneal loss, or blinks) were discarded (this was extremely rarely the case). To cope with variable level of noise in eye tracking recordings the following measures were taken. First, both thresholds were computed on a trial basis. Second, for each trial, potential shifts in eye position estimates were corrected by using the position of the fixation mark onto which subjects were explicitly asked to fixate before stimulus onset. Third, eye-tracker calibration was performed before each experimental block (fifty trials).
